# Integrative causal inference and predictive modeling reveal the iron-related gene SLC17A4 as a key biomarker in chronic rhinosinusitis

**DOI:** 10.3389/fimmu.2025.1645726

**Published:** 2026-01-14

**Authors:** Jiajia Lv, Feifei Jiang

**Affiliations:** Department of Otorhinolaryngology, The First Hospital of China Medical University, Shenyang, Liaoning, China

**Keywords:** chronic rhinosinusitis, iron metabolism, Mendelian randomization, SLC17A4, machine learning

## Abstract

**Purpose:**

To investigate whether iron metabolism exerts a causal influence on chronic rhinosinusitis (CRS) and to identify iron-related biomarkers and regulatory genes with diagnostic and therapeutic potential.

**Methods:**

A two-sample Mendelian randomization (MR) analysis was conducted using large-scale GWAS summary statistics for four iron-related traits and three nasal inflammatory diseases. Significant SNPs were mapped to proximal genes and analyzed via Gene Ontology (GO), KEGG pathway enrichment, and protein–protein interaction (PPI) network construction. Candidate gene expression was validated using the GSE69093 transcriptomic dataset and qRT-PCR in nasal mucosal tissues from CRS patients and healthy controls. Molecular docking simulations were performed to assess ligand interactions, and clinical association and machine learning models were applied to evaluate diagnostic relevance and predictive performance.

**Results:**

MR analysis identified transferrin saturation (TSAT) as a causal protective factor for CRS (OR = 0.9988, P = 0.014). Thirty-one genes were mapped from MR-associated SNPs, with SLC17A4 highlighted as a key candidate gene. Enrichment analysis indicated involvement in iron metabolism and inflammatory regulation. SLC17A4 expression was significantly downregulated in both GSE69093 and clinical qRT-PCR samples. TSAT and SLC17A4 levels showed strong inverse correlations with Lund-Mackay and SNOT-22 scores. Molecular docking identified Troglitazone as a strong-binding ligand to SLC17A4 (−10.0 kcal/mol). Machine learning models integrating iron biomarkers and SLC17A4 expression achieved high discriminative performance (AUC = 0.828–0.849) and demonstrated good calibration and net clinical benefit according to calibration and decision curve analyses, supporting their potential clinical applicability.

**Conclusion:**

TSAT confers protective effects in CRS, and SLC17A4 represents a promising biomarker and therapeutic target. The integrative strategy combining causal inference, transcriptomic validation, molecular docking, and machine learning modeling links iron homeostasis to CRS pathophysiology and demonstrates translational potential through clinically applicable predictive models.

## Introduction

Chronic rhinosinusitis (CRS) is a heterogeneous and multifactorial inflammatory disorder of the sinonasal mucosa that affects approximately 11–12% of adults worldwide, with regional variations ranging from 5% in East Asia to over 15% in parts of Europe and North America (1, 2). Characterized by persistent nasal obstruction, discharge, facial pressure, and olfactory dysfunction lasting more than 12 weeks, CRS poses a considerable burden on quality of life and is associated with sleep disturbances, depression, and impaired work productivity (3, 4). In the United States alone, direct healthcare costs related to CRS exceed $8 billion annually, highlighting the urgent need to elucidate its underlying pathophysiological mechanisms (5, 6).

In recent years, growing evidence has highlighted the critical role of systemic iron homeostasis in regulating immune function and inflammation (7). Iron serves as a cofactor in numerous enzymatic reactions and influences innate immune responses, including the generation of reactive oxygen species, cytokine production, and pathogen resistance (8). Dysregulated iron metabolism has been implicated in chronic inflammatory diseases such as inflammatory bowel disease, rheumatoid arthritis, and neuroinflammatory disorders (9, 10). However, the relationship between iron regulatory biomarkers and CRS pathogenesis has not yet been thoroughly investigated.

Genome-wide association studies (GWAS) have emerged as a powerful tool to identify genetic variants associated with complex traits and diseases (11, 12). Nevertheless, GWAS alone cannot establish causality due to potential confounding and reverse causation. Mendelian randomization (MR), which utilizes genetic variants as instrumental variables (IVs) to infer causal relationships between exposures and outcomes, offers a complementary approach that mitigates these limitations and has been increasingly applied in immunology and respiratory research (13, 14).

SLC17A4, a member of the solute carrier family 17, has recently attracted attention due to its putative role in phosphate and anion transport (15, 16). Preliminary transcriptomic evidence suggests that SLC17A4 expression is altered in CRS patients, yet its functional relevance and upstream regulatory mechanisms remain unclear (17). Notably, genetic loci linked to iron metabolism have been associated with variations in SLC17A4 expression, indicating a potential interface between iron homeostasis and CRS pathophysiology.

To address this gap, we conducted an integrative analysis combining two-sample MR, transcriptomic validation, retrospective clinical data analysis, and machine learning modeling. Our objectives were threefold (1): evaluate whether iron-related biomarkers causally influence CRS risk; (2) validate the expression and function of SLC17A4 in CRS; and (3) to construct predictive models incorporating iron-related biomarkers for potential clinical application. This study aims to provide novel insights into the mechanistic interplay between systemic iron regulation and CRS, and to identify SLC17A4 as a promising diagnostic and therapeutic target.

## Materials and methods

### Public dataset collection and processing

GWAS summary statistics for nasal diseases and iron-related biomarkers were retrieved from publicly accessible consortia. Data on allergic rhinitis (5,527 cases and 212,387 controls), chronic sinusitis (3,014 cases and 481,584 controls), and nasal polyps (2,019 cases and 460,914 controls) were sourced from the IEU Open GWAS project (https://gwas.mrcieu.ac.uk/), with each dataset comprising > 9 million SNPs. Summary statistics for serum iron (N = 163,511), transferrin saturation (TSAT, N = 131,471), total iron-binding capacity (TIBC, N = 135,430), and ferritin (N = 246,139) were obtained from the deCODE Genetics Consortium (https://www.decode.com/summarydata). Iron biomarkers were normalized using rank-based inverse normal transformation stratified by sex and adjusted for covariates, including age, menopausal status, ABO blood group, body mass index, smoking, alcohol intake, and iron supplementation, using generalized additive models. All GWAS datasets were harmonized to a reference genome build, and variants with ambiguous strand orientation or missing allele frequency information were excluded.

Transcriptomic data for nasal epithelial cells were obtained from the Gene Expression Omnibus (GEO) under accession number GSE69093. This dataset includes nasal epithelial samples from 13 patients with chronic rhinosinusitis and 11 matched healthy controls. Raw data were preprocessed using R software (v4.4.0), applying background correction, quantile normalization via the Robust Multi-array Average (RMA) method, and log2 transformation. Low-expressed probes were filtered out, and data quality was assessed through principal component analysis prior to downstream analysis.

### Mendelian randomization analysis

MR analyses were performed using the TwoSampleMR R package (version 0.5.7) to infer the potential causal relationship between iron-related biomarkers and nasal inflammatory diseases. SNPs significantly associated with iron traits (P < 5 × 10^-8^) were selected as IVs. If fewer than three IVs passed this threshold, a relaxed cutoff (P < 5 × 10^-6^) was used. LD clumping (r² < 0.001, 10,000 kb window) ensured IV independence, and IV strength was assessed via the F statistic (F > 10).

The primary MR analysis was employed using the inverse-variance weighted (IVW) approach under a fixed-effects model. Robustness was evaluated using the weighted median estimator and MR-Egger regression. Leave-one-out sensitivity analysis was performed, and horizontal pleiotropy was assessed via the MR-Egger intercept and MR-PRESSO (Mendelian Randomization Pleiotropy RESidual Sum and Outlier) global test. Reverse MR analysis was conducted by swapping exposure and outcome traits to test for bidirectionality.

### Target gene mapping and enrichment analysis

To functionally interpret the genetic variants implicated in the MR analysis, we annotated all IV SNPs used in the MR models. SNPs were mapped to proximal genes using the dbSNP database (https://www.ncbi.nlm.nih.gov/snp/), by locating genes within a ± 50 kb window around each SNP. After removing duplicates, a non-redundant gene list was compiled for enrichment analysis.

We employed the R package ClusterProfiler (version 4.6.2) to perform Gene Ontology (GO) and Kyoto Encyclopedia of Genes and Genomes (KEGG) enrichment analyses. The enrichment was carried out using default hypergeometric testing, with multiple testing correction applied via the Benjamini–Hochberg method (adjusted P < 0.05). GO biological processes related to iron homeostasis, inflammation, and epithelial regulation, as well as KEGG pathways involved in immune signaling and iron metabolism, were investigated. The output of these analyses was subsequently used to guide gene-level expression validation and downstream functional exploration.

### Molecular docking and drug interaction prediction

Molecular docking was conducted to predict potential ligand interactions with the protein product of the prioritized gene. Protein structure modeling was conducted using AlphaFold Protein Structure Database (https://alphafold.ebi.ac.uk/), using the full-length amino acid sequence to generate predicted 3D models in PDB format. Candidate small molecules were identified via keyword-based searches in the PubChem database (https://pubchem.ncbi.nlm.nih.gov/), focusing on compounds annotated to interact with the gene product according to existing literature or database annotations. Structures were downloaded in SDF or MOL2 formats and converted to PDBQT format using Open Babel (version 3.1.1).

Molecular docking simulations were performed using AutoDock Tools and AutoDock Vina (version 1.1.2) with binding regions defined around predicted active/transmembrane domains. Each compound was docked with an exhaustiveness of 8, and the top 10 poses were recorded. Binding affinities (kcal/mol) were averaged across three replicates. Results were visualized and inspected using PyMOL (version 2.5). No energy minimization or molecular dynamics simulations were applied at this stage.

### Clinical cohort and sample collection

A retrospective case-control study was conducted to validate the clinical relevance of iron metabolism-related biomarkers in CRS. Patients were recruited from the Department of Otolaryngology at The First Hospital of China Medical University between June 2022 and June 2023 In total, 183 patients diagnosed with CRS and 205 age- and sex-matched healthy controls were enrolled. All participants were ≥ 18 years old and provided written informed consent. The study was approved by the institutional ethics committee and conducted in accordance with the Declaration of Helsinki.

CRS was diagnosed based on the criteria defined by the European Position Paper on Rhinosinusitis and Nasal Polyps (EPOS 2020) and/or the American Academy of Otolaryngology–Head and Neck Surgery (AAO-HNS). Inclusion criteria required the presence of at least one cardinal symptom (nasal obstruction or discharge, anterior or postnasal), with or without facial pain/pressure or olfactory dysfunction, persisting for ≥ 12 weeks, and confirmed by endoscopic or radiological evidence. Exclusion criteria included: (1) prior nasal or sinus surgery; (2) recent use (within 3 months) of antibiotics or corticosteroids; (3) coexisting inflammatory or autoimmune diseases (e.g., rheumatoid arthritis, systemic lupus erythematosus); (4) hereditary or acquired disorders of iron metabolism (e.g., hemochromatosis); and (5) history of immunosuppressive therapy.

Nasal mucosal tissue specimens were collected from both CRS patients and control individuals during routine clinical procedures. For CRS patients, tissue samples were obtained during functional endoscopic sinus surgery, while for controls, tissues were harvested during corrective nasal surgeries (e.g., septoplasty or turbinate reduction) in individuals without a history of sinus disease. All samples were immediately preserved in RNAlater or snap-frozen in liquid nitrogen and stored at –80 °C until further RNA extraction and downstream molecular analyses.

### Laboratory assays and gene expression validation

Peripheral blood and clinical data were collected from each participant under standardized conditions. Collected indicators included age, sex, body mass index (BMI), C-reactive protein (CRP), white blood cell (WBC) count, and eosinophil percentage (EOS%). Iron-related biochemical markers, including serum iron, TSAT, ferritin, and hepcidin, were measured using automated chemiluminescent immunoassay and colorimetric methods according to the manufacturer’s instructions and institutional laboratory protocols.

Total RNA was extracted from nasal mucosal tissues using TRIzol reagent (Invitrogen, USA), and purity was assessed with a NanoDrop 2000 spectrophotometer (Thermo Fisher Scientific, USA). Reverse transcription was performed using the PrimeScript™ RT reagent kit (Takara, Japan). Quantitative real-time PCR (qRT-PCR) was carried out using the SYBR^®^ Premix Ex Taq™ II kit (Takara, Japan) on a LightCycler^®^ 480 instrument (Roche, Switzerland). Target genes included SLC17A4, SLC17A1, SCGN, and CARMIL1, with GAPDH serving as the endogenous reference. Amplification conditions were: 95°C for 30 s, followed by 40 cycles of 95°C for 5 s and 60°C for 30 s. All reactions were conducted in triplicate and expression levels were calculated using the 2^-ΔΔCt^ method. Primer sequences are listed in **Supplementary Table S1**.

### Machine learning model construction

To develop predictive models for CRS classification, clinical, biochemical, and molecular variables were used as candidate features. The Boruta algorithm, a random forest-based feature selection method, was employed to identify relevant variables. Data were split into training (80%) and test (20%) sets (N = 160/40). Five supervised algorithms—Random Forest (RF), XGBoost, CatBoost, Support Vector Machine (SVM), and Logistic Regression (LR)—were implemented using Python (version 3.10) and the scikit-learn framework. Grid search and Bayesian optimization (Optuna) were used for hyperparameter tuning. Performance was evaluated using the area under the receiver operating characteristic curve (AUC), along with accuracy, sensitivity, and specificity, with AUC serving as the primary evaluation metric.

### Clinical correlation and statistical analysis

Symptom-based and imaging-based severity scores were collected to evaluate the clinical relevance of iron metabolism markers and gene expression in CRS. The Lund-Mackay score quantified radiologic severity via CT, while SNOT-22 assessed symptom burden. Additional imaging metrics, including mucosal thickness and sinus opacification, were recorded. Spearman’s rank correlation was used to assess associations between iron biomarkers (TSAT, serum iron, ferritin, hepcidin), gene expression (e.g., SLC17A4), and clinical variables (Lund-Mackay, SNOT-22, CRP, EOS%). Continuous variables were compared between CRS and control groups using Student’s t-test or Mann–Whitney U test, and categorical data with Chi-square or Fisher’s exact test. Multicollinearity was evaluated via the variance inflation factor (VIF) before model construction. All statistical analyses were conducted in R (version 4.4.0), with two-sided P < 0.05 considered significant.

## Result

### Overview of the analytical framework

**Figure 1:**
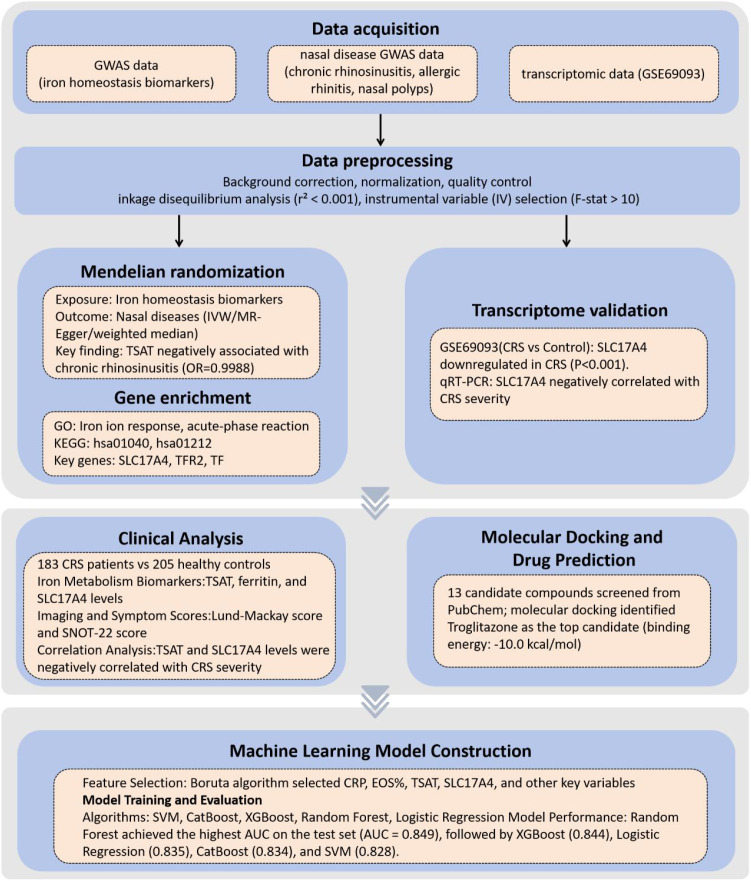
Schematic overview of the study design and analytical workflow.

We established a multi-step analytical framework to investigate the role of iron metabolism-related genes in CRS by integrating genomic, transcriptomic, clinical, and computational data. As illustrated in **Figure 1**, we first performed two-sample MR to evaluate the causal impact of systemic iron biomarkers on nasal inflammatory diseases using large-scale GWAS datasets. IVs were then mapped to proximal genes and analyzed through GO and KEGG pathway enrichment. To validate transcriptional relevance, gene expression levels were examined using the GSE69093 dataset and nasal tissue qRT-PCR assays. Clinical correlation analyses were performed to evaluate the associations between iron biomarkers and CRS severity scores. In parallel, we conducted molecular docking to explore potential compound–protein interactions. Finally, selected clinical and molecular features were incorporated into supervised machine learning models to predict CRS status, with model performance evaluated across five algorithms.

### Causal relationship between iron status and CRS based on MR

**Figure 2:**
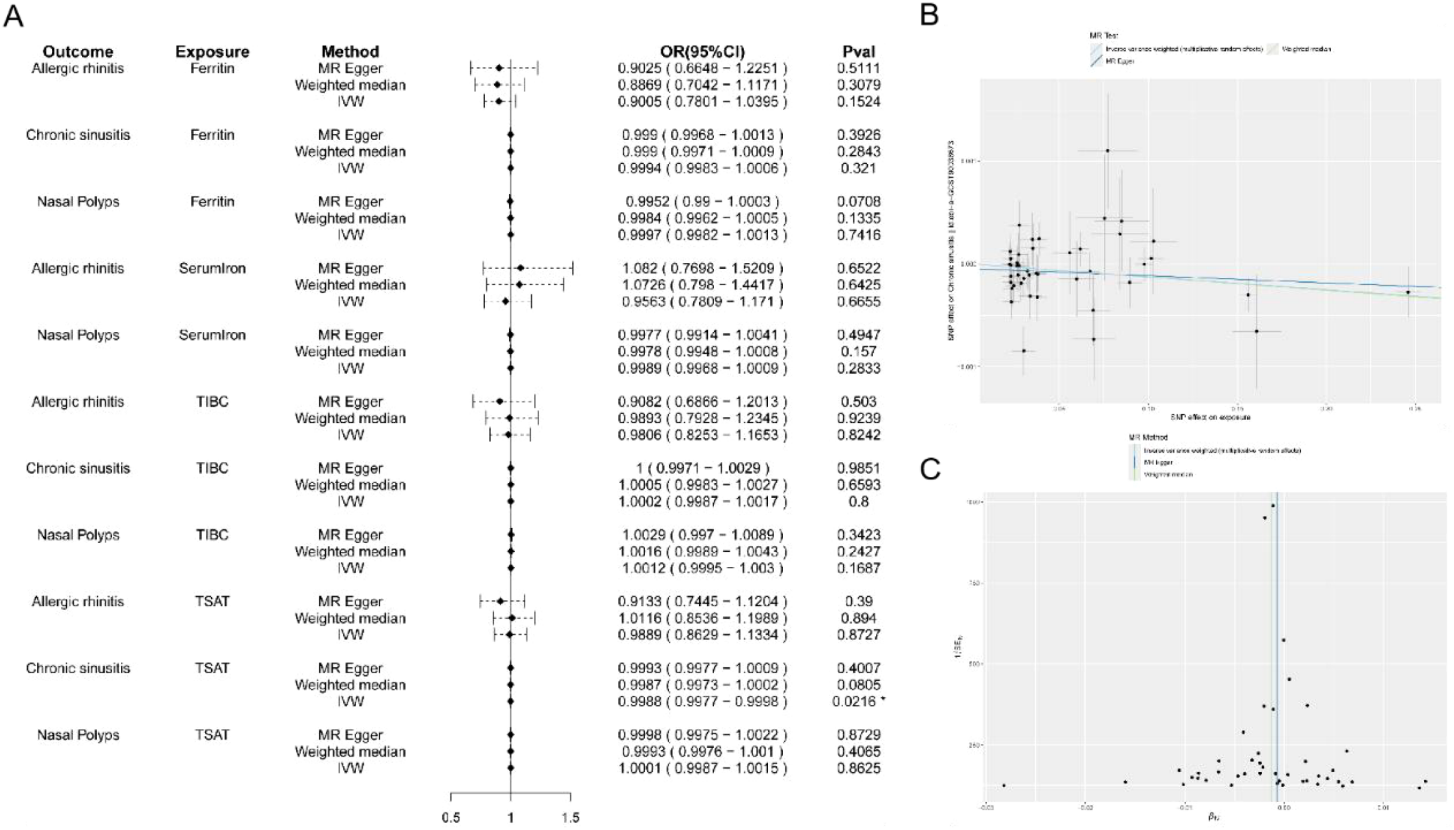
Mendelian randomization analysis evaluating the causal relationship between iron biomarkers and nasal inflammatory diseases. **(A)** Forest plot showing odds ratios (ORs) and 95% confidence intervals (CIs) for ferritin, serum iron, TIBC, and TSAT across three nasal diseases (allergic rhinitis, chronic sinusitis, and nasal polyps). **(B)** MR-Egger regression plot assessing potential horizontal pleiotropy for the TSAT–CRS association. **(C)** Funnel plot assessing heterogeneity in instrumental variable effects on TSAT and CRS outcomes.

To assess potential causal effects of systemic iron metabolism on nasal inflammatory diseases, we conducted two-sample MR analysis using summary-level GWAS data from large population cohorts (**Table 1**). Among the four iron-related biomarkers tested—ferritin, serum iron, TIBC, and TSAT—only TSAT showed a consistent and statistically significant inverse association with CRS. As shown in **Figure 2A**, the IVW method indicated a protective effect of TSAT on CRS risk (OR = 0.9988, 95% CI: 0.9980–0.9997, P = 0.0216), supported by MR-Egger and weighted median estimators. No significant associations were observed for TSAT with allergic rhinitis or nasal polyps, nor for ferritin, serum iron, or TIBC with any nasal diseases. Sensitivity analyses confirmed the robustness of the TSAT–CRS finding. The MR-Egger regression plot (**Figure 2B**) showed no evidence of horizontal pleiotropy, and the funnel plot (**Figure 2C**) revealed symmetry among IVs. Cochran’s Q statistic (Q = 47.603, P = 0.2552) and the MR-Egger intercept (P = 0.3804) indicated no significant heterogeneity or directional pleiotropy (**Table 2**), further supporting the validity of the IVs and the causal inference.

**Table 1:** Summary statistics of GWAS datasets used in the MR analysis.

Exposure (n)	Case	Control	Sample size	Number of SNPs
Allergic rhinitis	5,527	212,387	217,914	16,380,461
Chronic sinusitis	3,014	481,584	484,598	9,587,836
Nasal Polyps	2,019	460,914	462,933	9,851,867

**Table 2:** Heterogeneity and pleiotropy tests in MR analysis.

Outcome	Exposure	Heterogeneity		Pleiotropy
		Q	Q_pval	pval
Allergic rhinitis	Ferritin	245.6203	0.1648	0.9874
Chronic sinusitis		125.7274	0.5896	0.6758
Nasal Polyps		95.5102	0.1461	0.0736
Allergic rhinitis	Serum Iron	47.8676	0.3187	0.3805
Nasal Polyps		17.4922	0.5566	0.7126
Allergic rhinitis	TIBC	63.6981	0.0641	0.4963
Chronic sinusitis		61.1957	0.1134	0.8581
Nasal Polyps		21.2574	0.5654	0.5604
Allergic rhinitis	TSAT	45.2161	0.2284	0.3126
Chronic sinusitis		47.603	0.2552	0.3804
Nasal Polyps		32.1235	0.0569	0.7373

**Figure 3:**
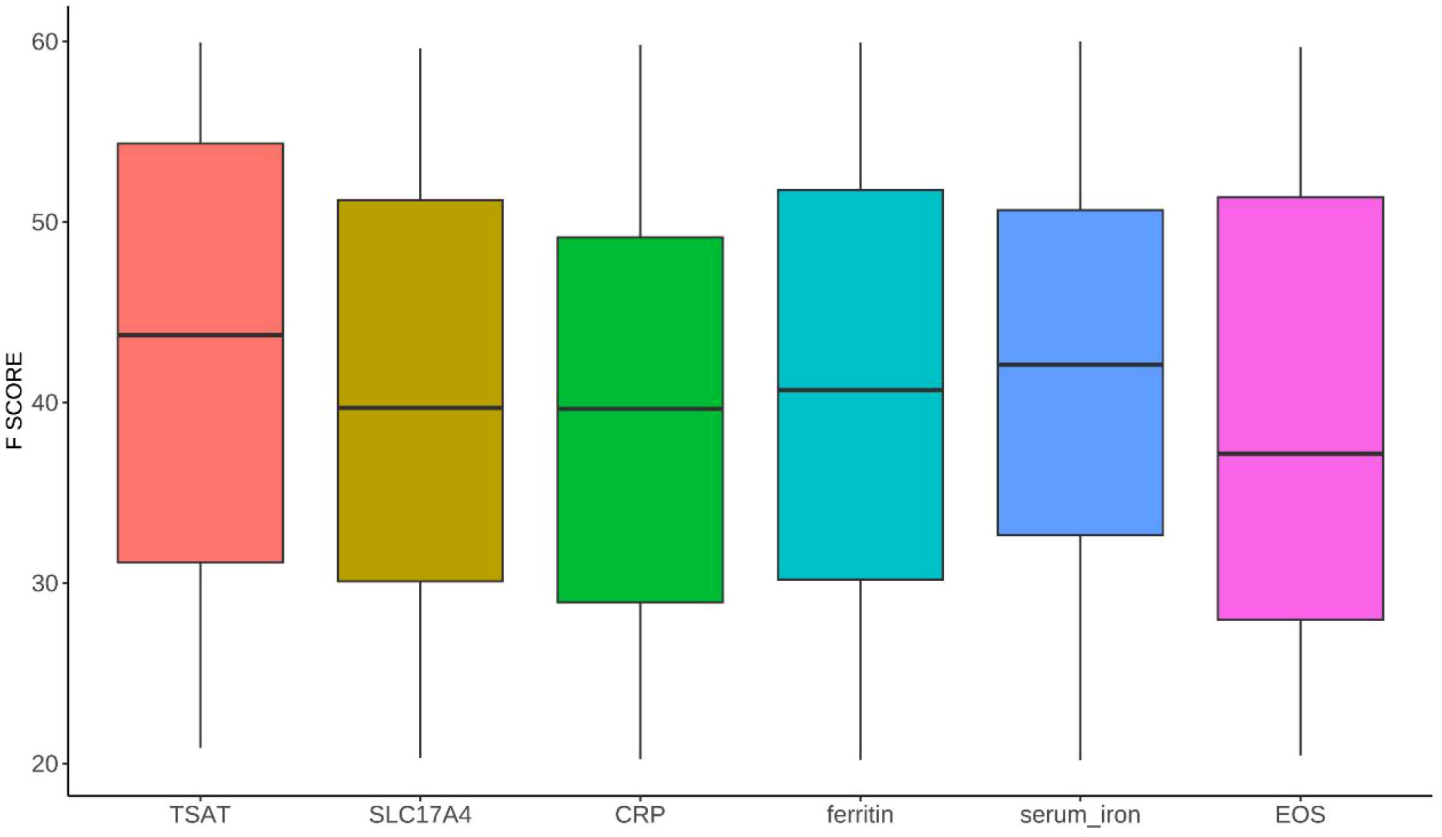
Distribution of F-statistics for instrumental variables associated with iron-related traits used in Mendelian randomization analysis. Boxplots display the range and median of F values for each exposure (TSAT, SLC17A4, CRP, ferritin, serum iron, and EOS).

To further ensure the robustness of causal inference, we evaluated the strength and validity of instrumental variables. The distribution of F-statistics indicated that all selected SNPs had F values > 10 (mean ≈ 30–50), suggesting no weak instrument bias (**Figure 3**). In addition, weak instrument sensitivity analysis using the MR-RAPS method produced results consistent with the IVW estimates across all outcomes, with a protective effect of TSAT on chronic sinusitis (OR = 0.69, 95% CI 0.46-0.88, P = 0.047) (**Table 3**).

**Table 3:** Sensitivity analysis for weak instrumental variables using the MR-RAPS method.

Outcome	Exposure	Method	nSNP	OR (95% CI)	P value
Allergic rhinitis	TSAT	Inverse variance weighted	29	0.77 (0.65–0.94)	0.025
Chronic sinusitis	TSAT	Inverse variance weighted	32	0.69 (0.46–0.83)	0.047
Nasal polyps	TSAT	Inverse variance weighted	41	0.78 (0.56–0.90)	0.038

### Functional annotation and network interaction of MR-linked target genes

**Figure 4:**
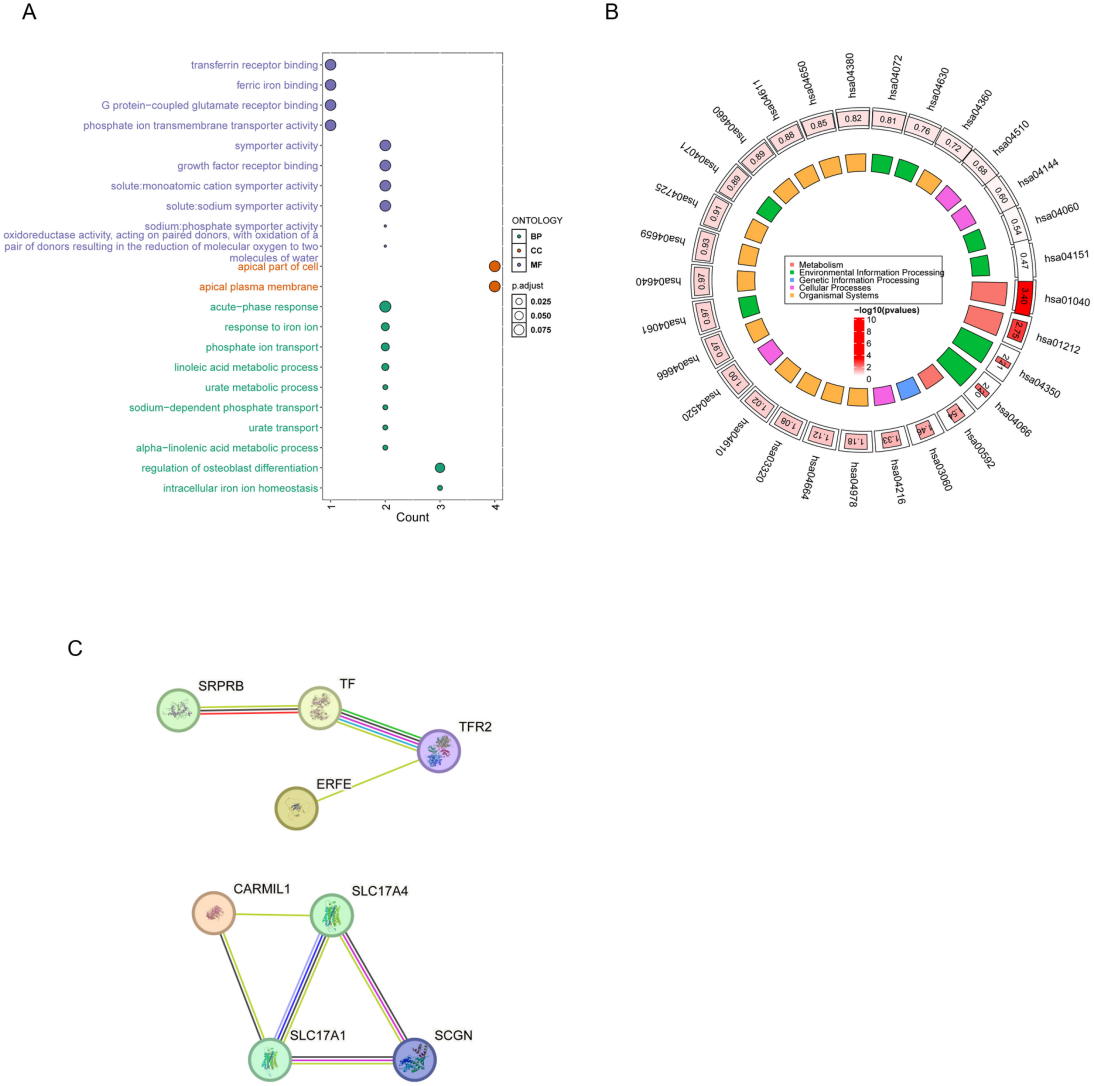
Enrichment and interaction analysis of TSAT-associated genes in CRS. **(A)** GO enrichment of 31 MR-linked genes showing overrepresentation in metal ion binding, transmembrane transport, and inflammatory pathways. **(B)** KEGG pathway enrichment highlights significantly enriched pathways such as ferroptosis, mineral absorption, and acute-phase response. **(C)** PPI network constructed via STRING database, revealing distinct interaction modules: one centered on SLC17A4, and another involving key iron-regulatory proteins (TF, TFR2, and ERFE).

Functional enrichment analysis was performed on 31 genes mapped from SNPs associated with the TSAT–CRS MR model. GO analysis revealed significant enrichment in molecular functions such as transferrin receptor binding, ferric ion binding, and transmembrane transporter activity, as well as biological processes including ion homeostasis and acute-phase response (**Figure 4A**). KEGG pathway enrichment identified key signaling pathways involved in ferroptosis, mineral absorption, and inflammatory responses (**Figure 4B**). Protein–protein interaction (PPI) network analysis showed two distinct clusters: one a SLC17A4-centered module involving SLC17A4, SLC17A1, CARMIL1, and SCGN, and another comprising iron-regulatory proteins including TF, TFR2, and ERFE (**Figure 4C**), highlighting the modular organization of iron metabolism-related genes in CRS pathophysiology.

### Baseline characteristics of the clinical cohort

A total of 183 patients with chronic rhinosinusitis (CRS) and 205 age- and sex-matched healthy controls were included. The baseline characteristics of both groups are summarized in **Table 4**. There were no significant differences in age, sex, or BMI between the two groups (all P > 0.05). However, CRS patients exhibited significantly higher levels of C-reactive protein (CRP), eosinophil percentage (EOS%), and white blood cell count (WBC) compared with controls (all P < 0.01). The proportion of individuals with a smoking history was also higher in the CRS group (P = 0.004). Among CRS patients, 61.7% were classified as CRS with nasal polyps (CRSwNP).

**Table 4:** Baseline characteristics of patients with chronic rhinosinusitis (CRS) and healthy controls.

Variable	CRS	Control	P_value
N	183	205	—
Age (years)	45.1 ± 11.4	45.5 ± 11.8	0.675
Gender (Male %)	93 (50.8%)	101 (49.3%)	0.839
BMI (kg/m2)	24.9 ± 3.2	24.7 ± 3.1	0.507
CRP (mg/L)	4.8 (3.4–6.7)	2.2 (1.5–3.0)	2.25e-39
EOS %	4.5 (3.3–5.4)	2.0 (1.5–2.9)	2.65e-42
Smoking history	80 (43.7%)	60 (29.3%)	0.004
WBC (10^9/L)	7.2 ± 1.4	6.4 ± 1.1	1.81e-09
CRSwNP %	113 (61.7%)	—	—

### Expression analysis of target genes in transcriptomic and clinical cohorts

**Figure 5:**
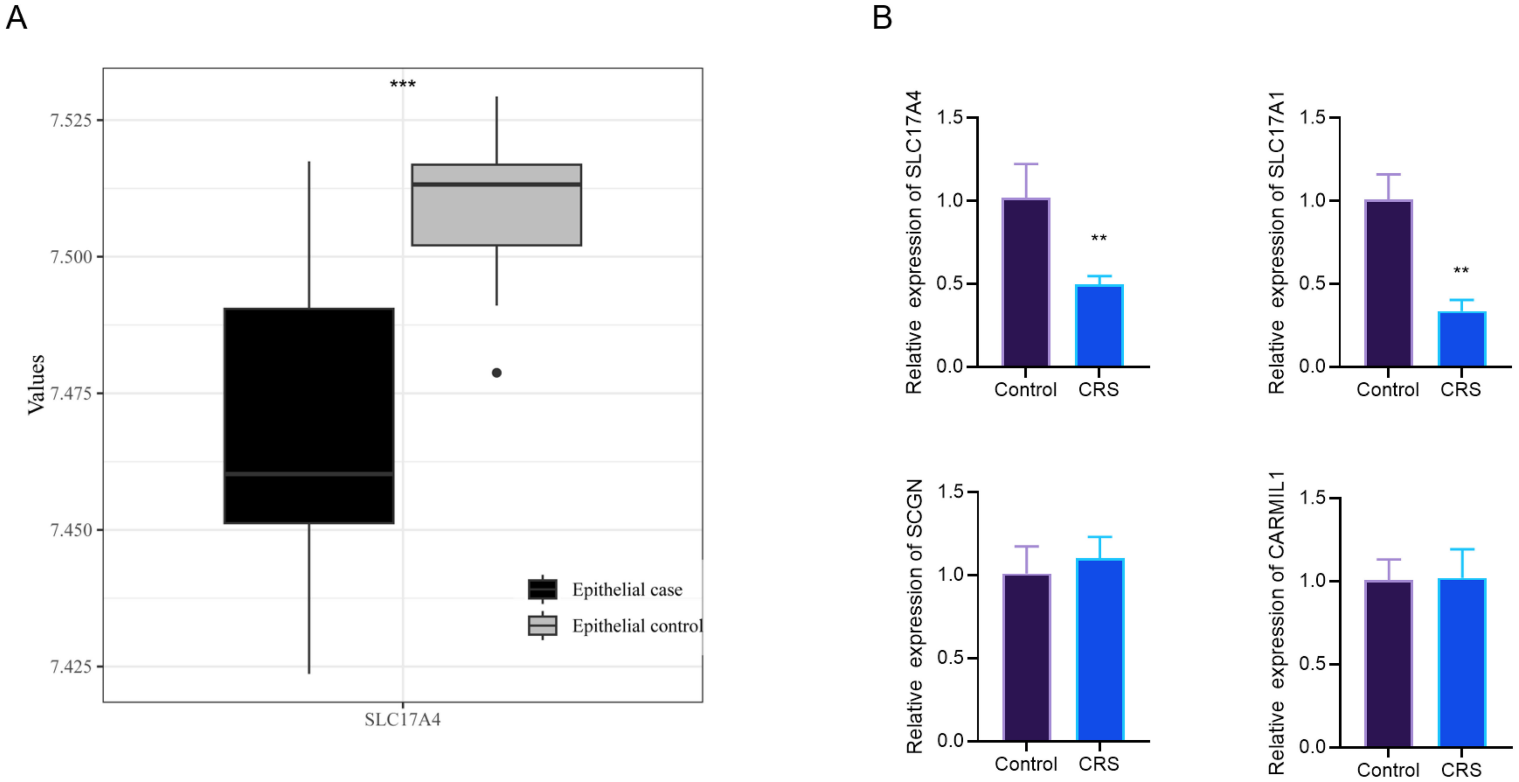
Expression analysis of MR-linked genes in nasal epithelial tissues. **(A)** Boxplot showing significantly reduced SLC17A4 expression in CRS patients versus controls based on the GSE69093 dataset. **(B)** Relative mRNA expression levels of SLC17A4, SLC17A1, SCGN, and CARMIL1 in CRS and control nasal tissues as assessed by qRT-PCR. ^*^P < 0.05, ^**^P < 0.01, ^***^P < 0.01 vs. Control.

To validate transcript-level alterations of MR-prioritized genes, we examined the expression of SLC17A4 in the nasal epithelium using both public datasets and clinical tissue samples. In the GSE69093 dataset, SLC17A4 expression was significantly lower in epithelial samples from CRS patients compared to controls (**Figure 5A**, P < 0.001). This finding was further confirmed by qRT-PCR analysis of nasal mucosal tissues, which demonstrated consistent downregulation of SLC17A4 in CRS patients. In addition, SLC17A1 expression was also significantly reduced (P < 0.01), while no significant differences were observed for SCGN and CARMIL1 (**Figure 5B**). Together, these results support the pathophysiological relevance of SLC17A4 in CRS and validate its transcriptomic and clinical signature across independent datasets.

### Molecular docking suggests troglitazone as a potential SLC17A4-interacting compound

**Figure 6:**
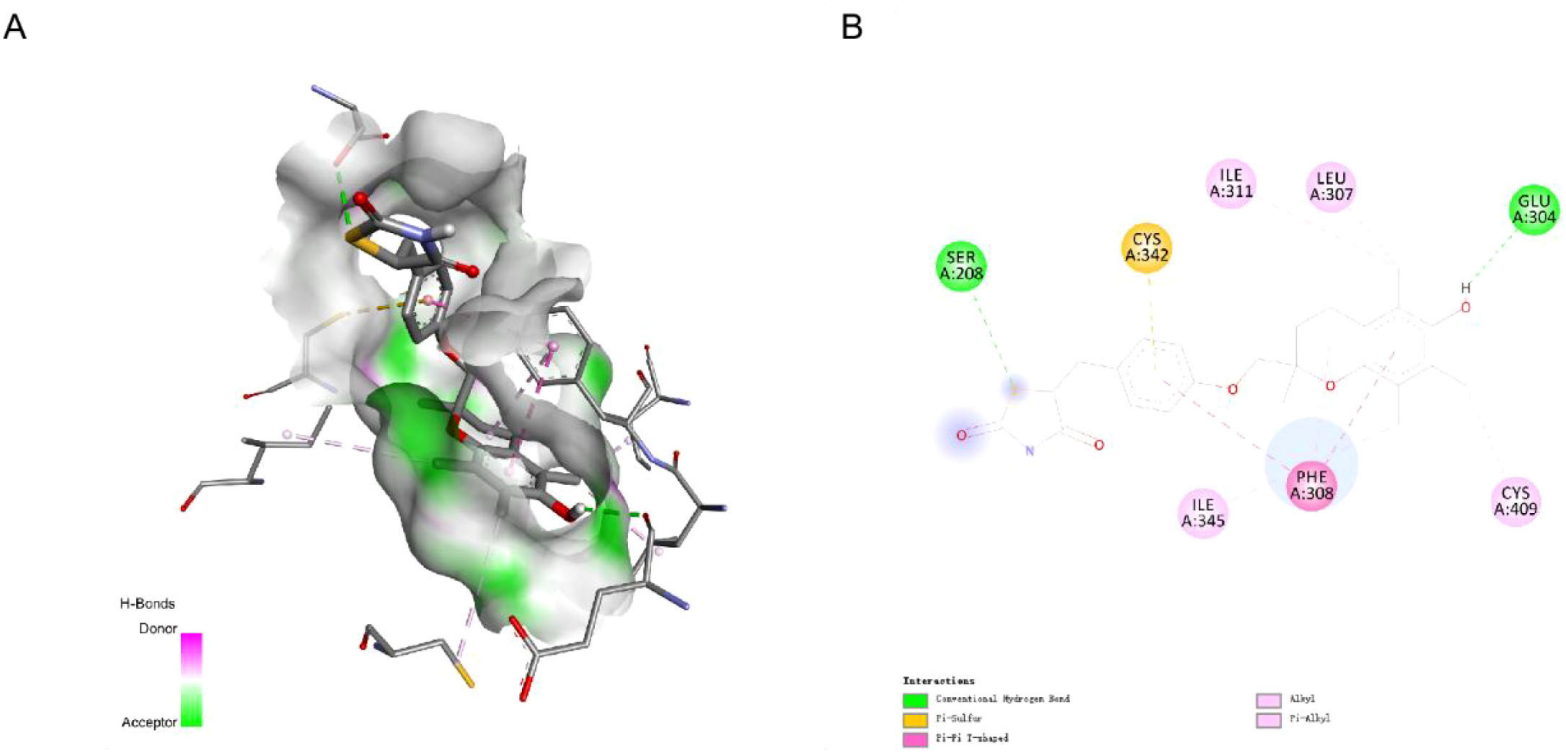
Molecular docking of Troglitazone with the SLC17A4 protein. **(A)** 3D docking conformation of Troglitazone bound to the predicted SLC17A4 structure. **(B)** 2D interaction map showing key residues involved in ligand binding, including conventional hydrogen bonds (green) and π-interactions (pink).

To explore the ligand-binding potential of SLC17A4, molecular docking was performed using compounds retrieved from PubChem with known or predicted interactions with solute carriers. Among the 13 candidate ligands, Troglitazone exhibited the strongest binding affinity to SLC17A4 (−10.0 kcal/mol), followed by Aflatoxin B1 (−9.9 kcal/mol) and Perfluorooctanesulfonic acid (−9.4 kcal/mol) (**Table 5**). The docking pose of Troglitazone revealed extensive molecular interactions within the predicted binding pocket, including conventional hydrogen bonding, π-alkyl stacking, and electrostatic interactions (**Figures 6A, B**). Key residues involved in stabilizing the complex included CYS342, PHE308, GLU304, and SER208, supporting the formation of a stable ligand–receptor interface. These findings suggest that Troglitazone may act as a potential modulator of SLC17A4, providing a chemical basis for further experimental validation in CRS-related therapeutic development.

**Table 5:** Predicted binding affinities of selected compounds to the SLC17A4 protein (AutoDock Vina).

Compound	Target	kcal/mol
Ethanol	SLC17A4	-2.6
Acetaminophen		-6.5
Valproic Acid		-5.7
Endosulfan		-5.9
Troglitazone		-10.0
Estradiol		-8.5
Bisphenol A		-7.6
2,3,7,8-Tetrachlorodibenzo-P-dioxin		-7.0
Silicon Dioxide		-2.1
Perfluorooctanesulfonic acid		-9.4
Fulvestrant		-8.6
Aflatoxin B1		-9.9
Cgp 52608		-6.3

### Iron biomarkers and gene expression correlate with CRS severity

**Figure 7:**
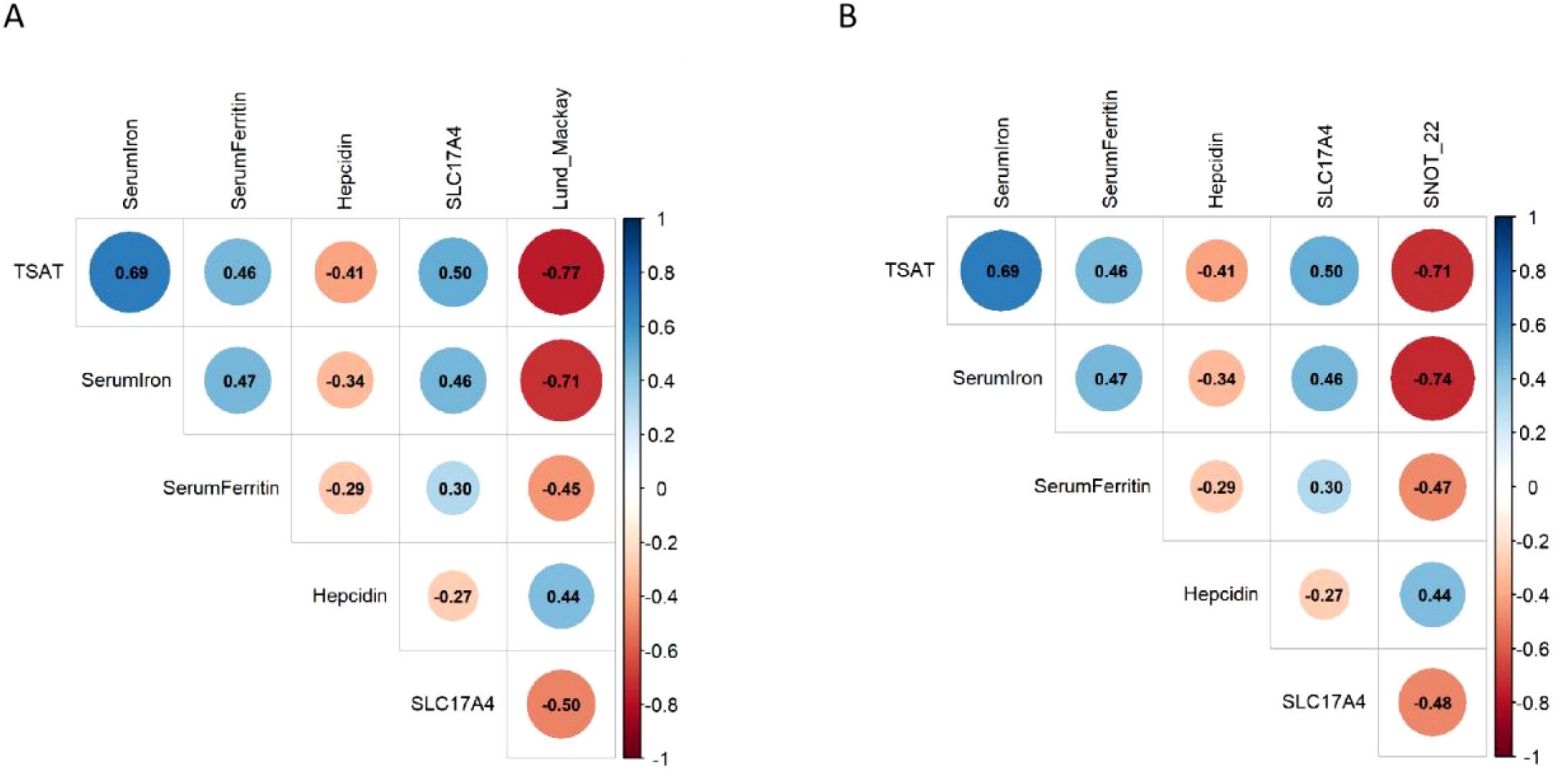
Correlation analysis between iron biomarkers, SLC17A4 expression, and clinical severity of CRS. **(A)** Spearman correlation coefficients between iron biomarkers and the Lund–Mackay score. **(B)** Spearman correlation coefficients between the same variables and the SNOT-22 score. Circle color indicates the strength and direction of correlation (blue = positive, red = negative); numeric values represent Spearman ρ coefficients.

To evaluate the relationship between iron metabolism and disease severity in CRS, Spearman correlation analysis was performed between iron-related biomarkers, SLC17A4 gene expression, and clinical severity scores. As shown in **Figure 7A**, both TSAT (ρ = −0.77) and SLC17A4 expression (ρ = −0.50) exhibited strong negative correlations with the Lund–Mackay score, a radiologic measure of disease burden. Consistent patterns were observed for SNOT-22 scores, with TSAT and SLC17A4 showing ρ = −0.71 and ρ = −0.48, respectively (**Figure 7B**), indicating that higher iron availability and SLC17A4 expression are associated with lower CRS symptom severity. In contrast, serum ferritin and hepcidin showed weak or nonsignificant correlations. These findings suggest that TSAT and SLC17A4 may serve as clinical biomarkers linked to disease progression and symptom severity in CRS.

### Machine learning model predicts CRS based on iron biomarkers and SLC17A4 expression

**Figure 8:**
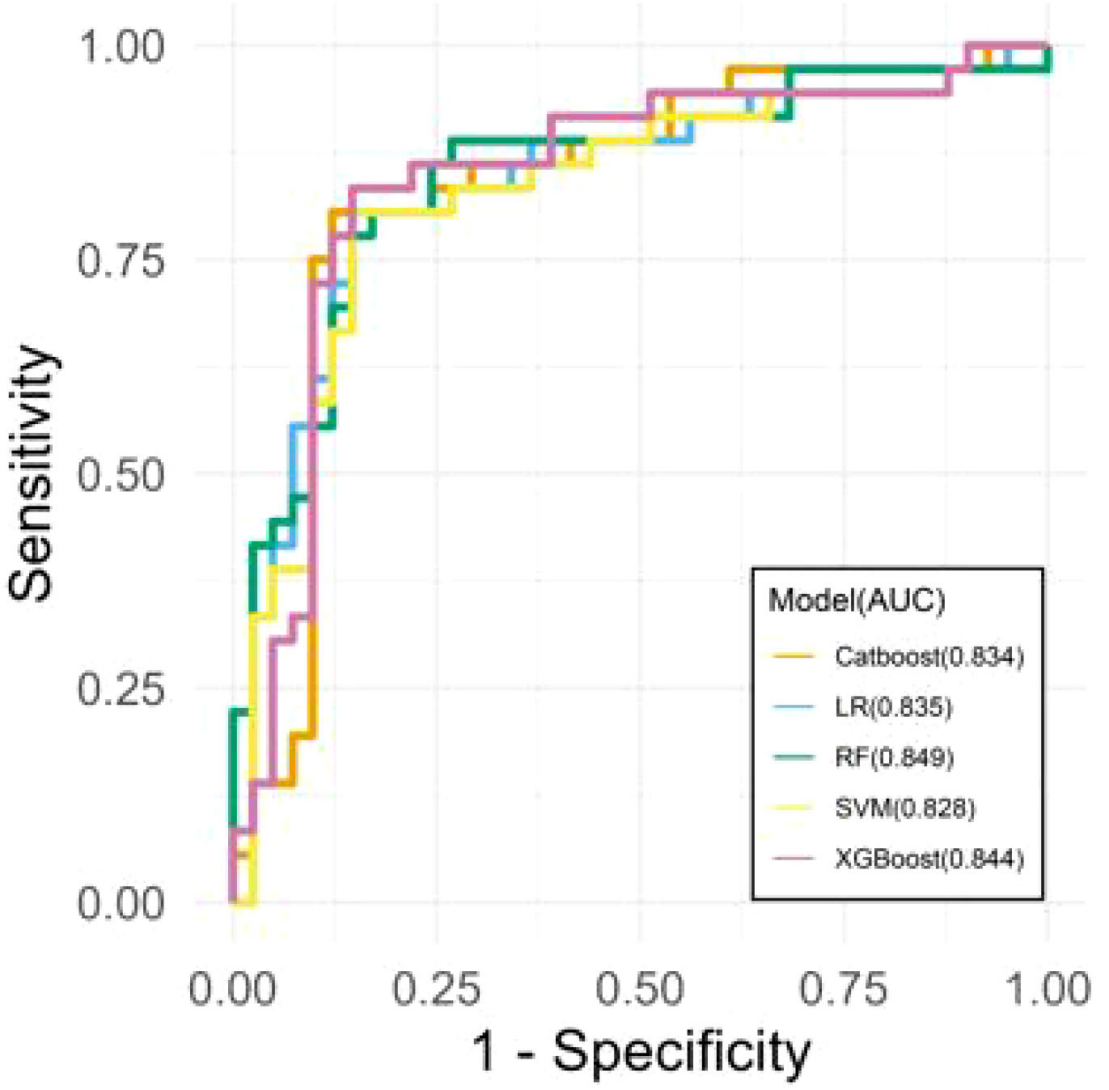
ROC curves of machine learning models predicting CRS based on iron metabolism features.

To evaluate the predictive potential of iron metabolism-related features in CRS diagnosis, five machine learning classification models were trained using features selected via the Boruta algorithm, including SLC17A4 expression, TSAT, serum iron, and inflammation-related markers. Models were evaluated on an independent test set (20% of the total cohort). As shown in **Figure 8**, all models achieved satisfactory classification performance, with AUC values ranging from 0.828 to 0.849. The RF model achieved the highest AUC (0.849), followed by XGBoost (0.844), LR (0.835), CatBoost (0.834), and SVM (0.828). These results indicate that iron homeostasis biomarkers and SLC17A4 expression jointly contribute to the accurate discrimination of CRS patients from healthy controls, supporting their potential role in biomarker-guided diagnostic modeling.

**Figure 9:**
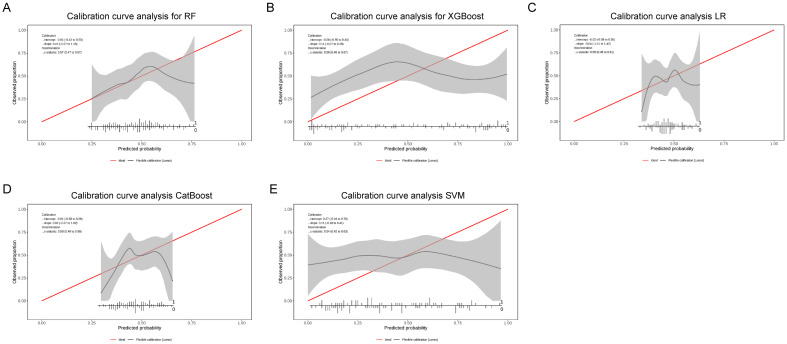
Calibration curves of machine learning models predicting CRS based on iron metabolism features. **(A)** Random Forest (RF), **(B)** XGBoost (XGB), **(C)** Logistic Regression (LR), **(D)** CatBoost, and **(E)** Support Vector Machine (SVM). The red diagonal line represents perfect calibration, while the black line indicates the model-predicted probabilities with 95% confidence intervals (gray shading).

**Figure 10:**
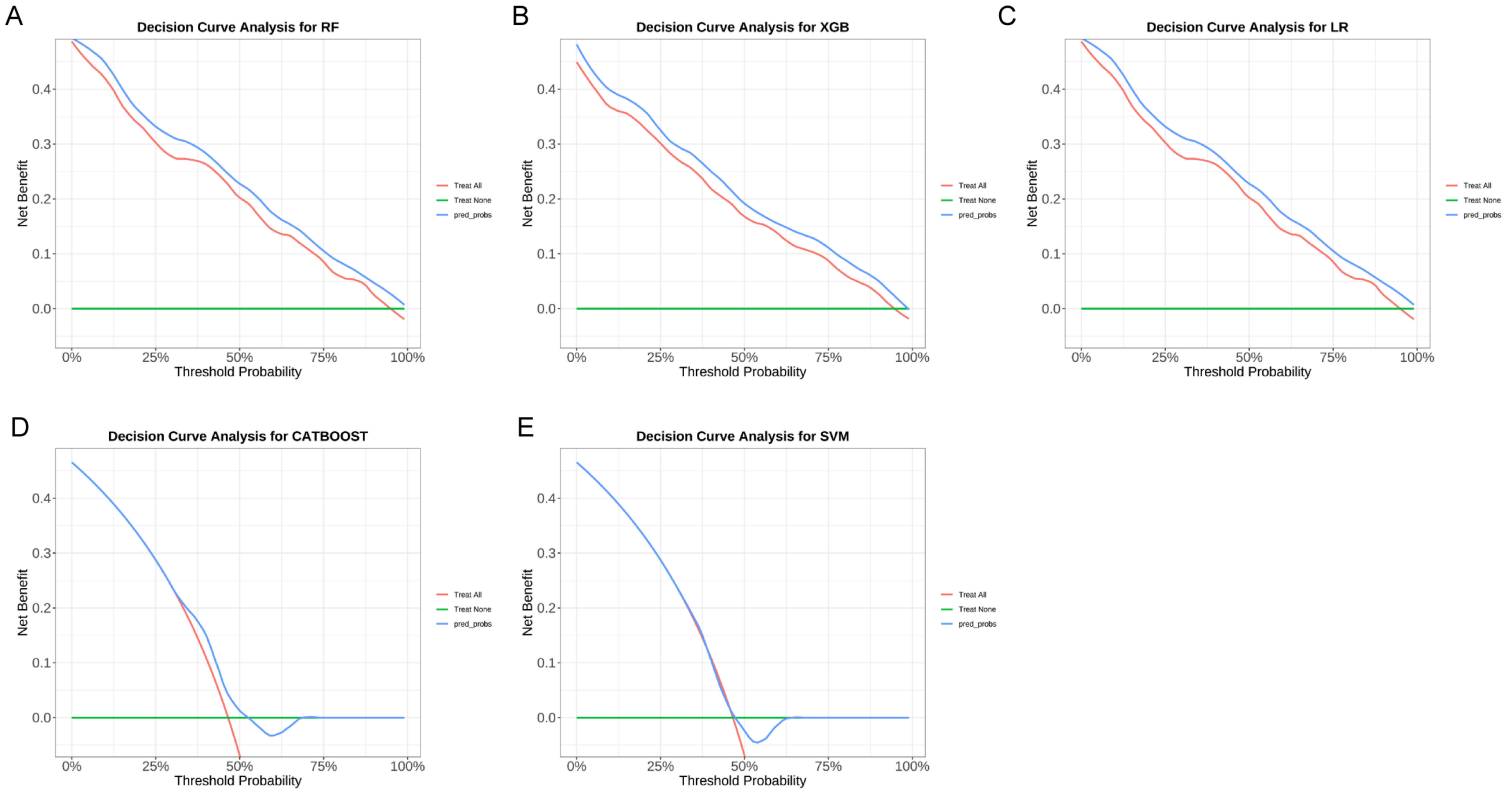
Decision curve analysis (DCA) of machine learning models for clinical utility assessment. **(A)** Random Forest (RF), **(B)** XGBoost (XGB), **(C)** Logistic Regression (LR), **(D)** CatBoost, and **(E)** Support Vector Machine (SVM). The x-axis represents threshold probability, and the y-axis indicates net benefit. The red line represents the model, the blue line represents the “treat-all” strategy, and the green line represents the “treat-none” strategy. Models demonstrating higher net benefit across a wide range of threshold probabilities indicate superior clinical usefulness.

To further evaluate the clinical applicability of the predictive models, calibration and decision curve analyses were conducted. As shown in **Figure 9**, the calibration curves demonstrated good agreement between the predicted probabilities and observed outcomes across all five models, with the Random Forest (RF) and XGBoost (XGB) models showing the best calibration performance. In addition, **Figure 10** presents the decision curve analysis (DCA) results, indicating that the inclusion of iron metabolism biomarkers and SLC17A4 expression provided a higher net clinical benefit across a wide range of threshold probabilities compared to the “treat-all” and “treat-none” strategies. These findings suggest that the predictive models not only exhibit high discriminative ability (AUC) but also possess favorable calibration and clinical utility, supporting their potential for clinical translation.

Models with curves closer to the diagonal demonstrate better agreement between predicted and observed probabilities.

In addition to AUC, multiple classification metrics were evaluated to comprehensively assess model performance, including sensitivity, specificity, precision and recall. As summarized in **Table 6**, all five machine learning models demonstrated stable and satisfactory performance. The Random Forest (RF) model achieved the best overall performance (AUC = 0.849, sensitivity = 0.80, specificity = 0.78, precision = 0.77, recall = 0.80), with consistent 5-fold cross-validation AUC values of 0.841, indicating robust generalization capability.

**Table 6:** Classification performance metrics and five-fold cross-validation results of machine learning models for predicting chronic rhinosinusitis (CRS).

Model	AUC	Sensitivity	Specificity	Precision	Recall	5-fold CV AUC
RF	0.849	0.80	0.78	0.77	0.80	0.841
XGBoost	0.844	0.78	0.80	0.76	0.78	0.839
LR	0.835	0.75	0.82	0.74	0.75	0.826
SVM	0.828	0.72	0.85	0.70	0.72	0.813
CatBoost	0.834	0.76	0.81	0.75	0.76	0.822

To further enhance interpretability, feature importance analysis was performed using SHAP (Shapley Additive Explanations) values and variable importance scores. As shown in **Table 7**, TSAT and SLC17A4 were the most influential predictors across all models, followed by CRP, ferritin, and serum iron. These findings indicate that both systemic and molecular iron markers contribute substantially to CRS classification and prediction accuracy.

**Table 7:** Feature importance ranking of variables contributing to model prediction across five machine learning algorithms.

Feature	RF	XGBoost	LR	SVM	CatBoost
TSAT	24.52	0.95	1.16	6.25	19.62
SLC17A4	23.43	0.70	0.97	4.51	18.21
CRP	23.24	0.67	0.70	4.13	17.87
EOS	22.33	0.65	0.68	3.77	17.80
ferritin	21.63	0.64	0.67	3.47	17.79
serum iron	21.28	0.64	0.34	1.00	14.27

## Discussion

This study applied a multi-omics, integrative framework to investigate the role of iron metabolism—particularly TSAT—in CRS. We identified TSAT as a potential protective factor against CRS through MR, supported by transcriptomic and qRT-PCR validation of the downregulation of SLC17A4, a gene mapped from TSAT-associated SNPs. Clinical correlation analysis further showed that both TSAT levels and SLC17A4 expression were inversely associated with CRS severity. Molecular docking suggested that Troglitazone may bind stably to SLC17A4, and a machine learning model based on iron biomarkers and gene expression demonstrated strong predictive power for CRS status.

Although CRS has been extensively studied in terms of epithelial dysfunction, microbiota imbalance, and local immune responses (18), the potential contribution of systemic iron metabolism has received little attention. Our MR analysis provides the first causal evidence linking systemic iron availability, as reflected by TSAT, to reduced CRS risk, expanding prior observations that associate iron deficiency with impaired immune resilience and heightened inflammatory susceptibility (19). Several chronic inflammatory conditions, such as inflammatory bowel disease and rheumatoid arthritis, have shown clinical associations with altered iron handling, particularly via TSAT and hepcidin regulation (20). In contrast to hepcidin or ferritin, which are acute-phase reactants, TSAT reflects iron availability in circulation and correlates more directly with iron utilization at the tissue level. Our finding that TSAT correlates negatively with both Lund-Mackay and SNOT-22 scores suggests a potential protective role of systemic iron sufficiency in maintaining mucosal homeostasis in CRS, possibly through modulation of epithelial redox balance and immune tone. Mechanistically, iron availability regulates macrophage polarization, T-cell activation, and cytokine secretion, thereby shaping the inflammatory milieu and influencing epithelial repair and immune tolerance (21) SLC17A4, previously studied in the context of intestinal phosphate transport, has not been linked to airway disease. However, its structural homology to SLC17A1, a gene involved in urate and inflammation pathways, and its consistent downregulation in CRS, point to a potential functional role in mucosal iron regulation (22). In our study, the downregulation of SLC17A4 in CRS mucosa, along with its strong inverse correlation with TSAT and disease severity, points to its potential as a key mediator in iron–epithelial signaling. PPI network analysis revealed that SLC17A4 clusters with TFR2, TF, and ERFE, all of which are well-established regulators of systemic iron homeostasis (23), suggesting that SLC17A4 might function at the interface of local and systemic iron regulation. Our docking analysis further supports the translational potential of SLC17A4 by identifying Troglitazone as a compound with high binding affinity. Troglitazone, a known PPARγ agonist, has demonstrated anti-inflammatory effects in airway models by stabilizing epithelial barriers and reducing cytokine production (24). The predicted interaction between Troglitazone and SLC17A4 suggests that PPARγ-mediated signaling may intersect with iron transporter pathways, offering a mechanistic link worthy of further exploration. In contrast to prior CRS biomarker studies that focus on tissue eosinophilia, IL-5, or remodeling markers (25), this study brings a novel systemic metabolic dimension to the disease. By combining genomics, transcriptomics, biochemical data, and machine learning, we demonstrate that SLC17A4 and TSAT are not only biologically relevant but also clinically actionable markers, with potential for stratifying patient subgroups and guiding treatment decisions.

The biological mechanisms underlying the TSAT–SLC17A4–CRS axis remain to be fully elucidated. Our findings demonstrated that SLC17A4 expression was significantly downregulated in lesioned nasal tissues and negatively correlated with both Lund–Mackay and SNOT-22 scores, suggesting that reduced SLC17A4 expression may contribute to disease severity. Future studies involving SLC17A4 knockdown or overexpression models are warranted to elucidate its functional role in epithelial and iron-mediated inflammatory regulation. One possibility is that iron sufficiency maintains SLC17A4 expression in the nasal epithelium, contributing to proper ion transport, oxidative stress control, or mucosal defense. In conditions of iron deficiency, suppression of SLC17A4 may impair epithelial resilience, increasing susceptibility to chronic inflammation. Alternatively, SLC17A4 may serve as a downstream effector of TSAT-regulated pathways that modulate epithelial gene expression or innate immunity. Although the precise mechanism through which systemic iron availability (TSAT) regulates SLC17A4 expression remains to be determined, previous studies suggest that iron-dependent transcription factors such as HIF-1α, IRP1/2, or PPARγ may mediate this interaction (26) Given that SLC17A4 exhibits homology to other solute carriers responsive to metabolic and redox cues, future work will focus on elucidating whether TSAT influences SLC17A4 through these signaling pathways. From a clinical perspective, the identification of TSAT and SLC17A4 as correlated with both CRS risk and severity suggests their utility as diagnostic or prognostic biomarkers. Unlike conventional inflammatory markers such as CRP, IL-5, periostin, or eosinophil counts—which primarily indicate local inflammatory activity—SLC17A4 reflects systemic iron homeostasis and epithelial transport function, representing a metabolically driven regulatory axis. This distinction underscores its novelty and potential to complement existing CRS biomarkers by integrating immune and metabolic dimensions. The robust performance of machine learning models further highlights the feasibility of incorporating molecular and metabolic features into CRS prediction tools. The calibration and decision curve analyses further confirmed the reliability and potential clinical applicability of these models, underscoring their translational relevance in CRS risk prediction. Furthermore, the identification of Troglitazone as a potential SLC17A4 modulator provides a starting point for therapeutic repurposing or lead compound development targeting mucosal iron regulation. Future studies should include SLC17A4 knockdown or overexpression in nasal epithelial cells and animal models to validate its functional role in iron-dependent signaling and mucosal immune regulation.

Despite the strengths of our integrative approach, several limitations warrant consideration. First, the MR analysis, although powerful in inferring causality, relies on assumptions regarding instrument validity and absence of pleiotropy. Second, functional validation of SLC17A4 in epithelial models and its response to TSAT manipulation were not performed in this study. Third, docking simulations, while useful for screening, must be complemented by binding assays and cellular experiments. Finally, the clinical cohort was drawn from a single center and warrants replication in diverse populations. Future studies should include mechanistic experiments (e.g., SLC17A4 knockdown or overexpression in epithelial cells), animal models, and prospective validation of prediction models across populations and disease subtypes. Additionally, the impact of iron supplementation or modulation of PPARγ signaling on CRS outcomes could offer novel therapeutic avenues.

## Conclusion

This study uncovers a previously unrecognized connection between systemic iron homeostasis and CRS pathogenesis. Through MR analysis, we demonstrate that transferrin saturation (TSAT) is causally protective against CRS, and that SLC17A4 is downregulated in diseased tissues and correlates inversely with symptom severity. The integrative strategy employed here, combining causal inference, transcriptomic validation, molecular modeling, and clinical prediction, highlights SLC17A4 as a novel biomarker and potential therapeutic target in CRS.

## Data Availability

The original contributions presented in the study are included in the article/**Supplementary Material**. Further inquiries can be directed to the corresponding author.

## Supplementary material

Supplementary Table 1 Primer sequence list
