# Supplementary material for: Integrative causal inference and predictive modeling reveal the iron-related gene SLC17A4 as a key biomarker in chronic rhinosinusitis
Source: Front Immunol. 2026 Jan 14;16:1645726. doi: 10.3389/fimmu.2025.1645726 (PMC12847307; doi:10.3389/fimmu.2025.1645726)
Supplement: Supplementary Table 1 — Primer sequence list [file Table1.docx]

**Table legends：**

Table S1.Primer sequence list

|  | Forward primer | Reverse primer |
| --- | --- | --- |
| SLC17A4 | ACCAAAATGTCTACCGGACC | ATCCCTGTGGTTGAGTCCAT |
| SLC17A1 | TCCGGAATCCGCCTGGTTTA | GCTCTGCAAGTGCTCTGTTT |
| SCGN | GTCCTCCCCAGCAACAGTTA | GGTGTTGACGACGAAGACCA |
| CARMIL1 | AGAGAAGAGAGCTGCGTGTG | CTGGGTGCAGTTTAGGCACTT |
